# Telitacicept as double-targeted therapy for myasthenia gravis coexisting with connective tissue disease: three case reports

**DOI:** 10.3389/fimmu.2025.1552521

**Published:** 2025-05-27

**Authors:** Yingying Yang, Ying Zhu, Ruixia Zhu

**Affiliations:** ^1^ Department of Neurology, The First Affiliated Hospital of China Medical University, Shenyang, China; ^2^ Key Laboratory of Neurological Disease Big Data of Liaoning Province, Shenyang, China; ^3^ Shenyang Clinical Medical Research Center for Difficult and Serious Diseases of the Nervous Systems, Shenyang, China

**Keywords:** myasthenia gravis, connective tissue disease, concomitant, telitacicept, case report

## Abstract

Myasthenia gravis (MG) and connective tissue diseases (CTD) are both B-cell-mediated, antibody-associated autoimmune diseases that share similar mechanisms of immune dysfunction. The coexistence of MG and CTD is a rare phenomenon, and its management remains challenging. Here, we report three cases of coexisting MG and CTD—specifically systemic lupus erythematosus and Sjögren’s syndrome, and all three patients presented significant improvement 4 weeks after initiation of treatment with telitacicept. Minimal symptom expression (MSE) was achieved after 4, 6, and 7 weeks of treatment with telitacicept, for patients one-to-three, respectively. This therapy also enabled a reduction in prednisone dosage, with clinical symptoms of CTD remaining well controlled. These findings present preliminary evidence supporting Telitacicept as an effective treatment and a double-target therapy for the management of MG-CTD.

## 1 Introduction

Myasthenia gravis (MG) is a B-cell-mediated, antibody-associated, and biologically complex autoimmune disease characterized by skeletal muscle weakness that worsens with activity. Autoimmune diseases (ADs) are more likely to co-occur than expected by chance. Connective tissue diseases (CTD), including rheumatoid arthritis, scleroderma, Sjögren’s syndrome (SS), and systemic lupus erythematosus (SLE), have been reported to coexist with MG (1–4). Notably, both MG and CTD are chronic immune-mediated conditions, with the incidence of concomitant ADs in patients with MG estimated at approximately 13%. Thyroid disorders, SLE, and vitiligo are among the most common systemic ADs (5). Therefore, investigating the underlying mechanisms of MG-CTD is urgently needed to identify optimal treatment strategies. ADs share common pathogenic mechanisms. When B cells were stimulated by follicular helper T cells, the autoimmune antibodies, such as acetylcholine receptor antibodies (AChR-Ab) in MG and anti-dsDNA antibodies in SLE, will be secreted by activated B cells (6). This process highlights the central role of B cells in autoimmune pathogenesis. B lymphocyte stimulating factor (BLyS) and a proliferation-inducing ligand (APRIL) are significant for the activation, development and survival of B cells (7). Consequently, selecting telitacicept, a targeted immunosupression on both of BLyS and APRIL, might be a suitable therapeutic strategy for MG-CTD. This report presents two patients initially diagnosed with MG who subsequently met the diagnostic criteria for CTD, along with one patient for whom the reverse sequence of diagnoses occurred. All three cases were successfully managed with telitacicept.

## 2 Case presentation

### 2.1 Case 1

**Figure 1:**
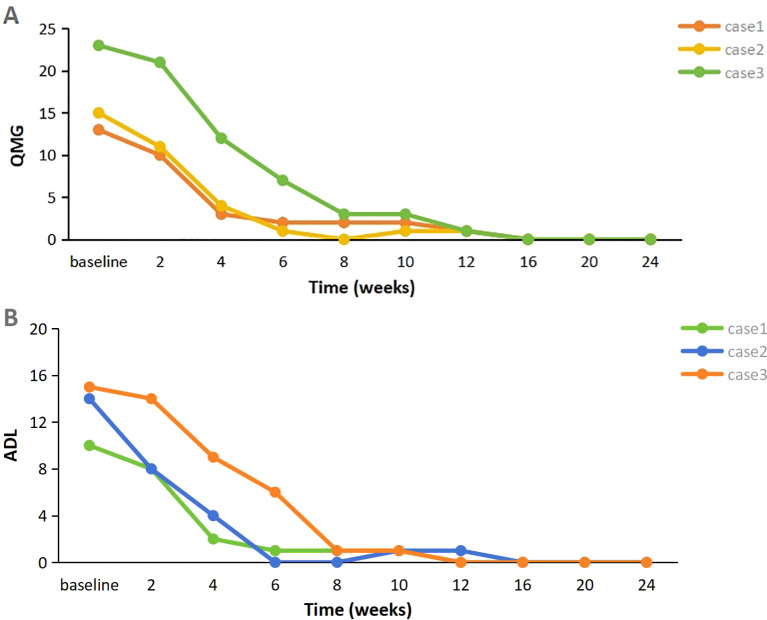
**(A, B)** Evolution of clinical severity of MG in three patients, assessed through the changes of QMG score and MG-ADL score (Baseline represents the time of initial use of telitacicept). MG-ADL myasthenia gravis specific activities of daily living scale; QMG quantitative myasthenia gravis score.

**Figure 2:**
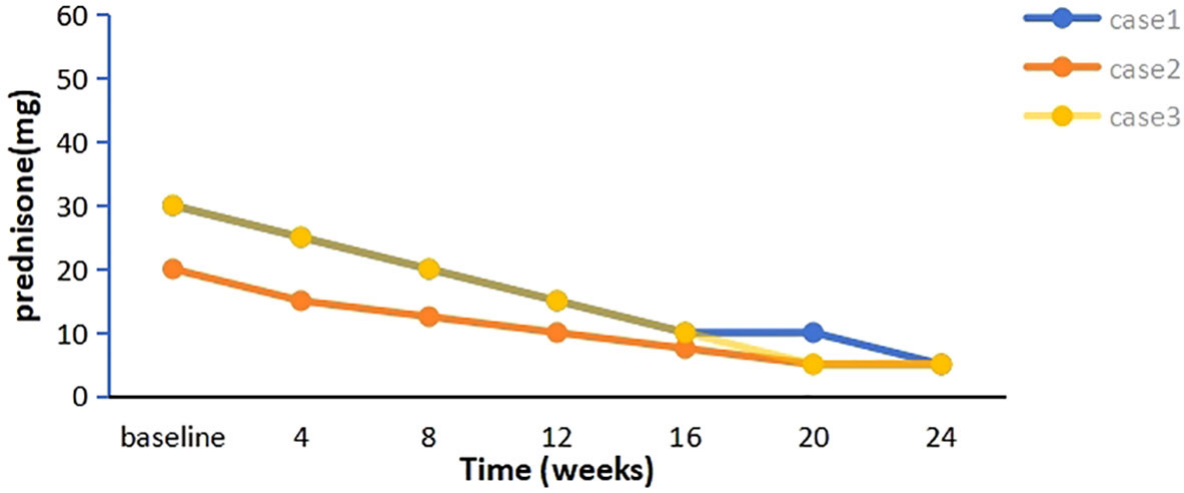
The tapering process of prednisone in three patients over 24 weeks.

A 58-year-old woman presented with a 2-year history of SLE, characterized by metacarpophalangeal and proximal interphalangeal joint pain, proteinuria, hematuria, anemia, leukopenia, high titers of antinuclear antibodies (ANA) and anti-dsDNA antibodies, and decreased complement C3 and C4 levels. She was treated with oral prednisolone (20 mg/d), hydroxychloroquine (200 mg twice daily), and leflunomide (20 mg/d), which resulted in improvement. Approximately one year later, she developed ptosis and diplopia that worsened throughout the day. Over time, she experienced generalized muscle weakness, dysphagia, dysarthria, and paroxysmal dyspnea. Involvement of neuromuscular junction was suspected. Both AChR-Ab and Titin antibody (Titin-Ab) levels were positive (0.88 nmol/L and 122.58 U/mL, respectively). A repetitive nerve stimulation (RNS) test showed a decremental response, and chest computed tomography (CT) was normal. Based on these clinical and laboratory findings, a diagnosis of generalized MG (gMG) (Myasthenia Gravis Foundation of America IIIb, MGFA IIIb) was established. The patient was treated with pyridostigmine (240 mg/d) but showed a poor response. Upon admission to our hospital, her Quantitative Myasthenia Gravis (QMG) and MG-activities of daily living (MG-ADL) scores were 13 and 10, respectively. Due to the co-occurrence of gMG and SLE, the patient was treated with telitacicept (160 mg/week). Two weeks after the initial dose, notable clinical improvement was observed, with QMG and MG-ADL scores decreasing to 10 and 8, respectively, particularly in dysphagia and dysarthria. After six weeks of treatment, her QMG and MG-ADL scores further improved further to 2 and 1, respectively (**Figure 1**), and she achieved minimal symptom expression (MSE), defined as an MG-ADL score of 0 or 1. Joint pain resolved, and laboratory parameters, including routine urine tests, blood counts, and complement levels, normalized. The prednisone dose was tapered from 30 mg/d to 5 mg/d and has been maintained (**Figure 2**). She has sustained MSE for six months, and her SLE activity has improved, achieving a mildly active state according to the SLE Disease Activity Index 2000 (SLEDAI-2K).

### 2.2 Case 2

A 28-year-old woman experienced palpitations and hand tremors for two months and was diagnosed with hyperthyroidism, for which she received thyrozol (15 mg/d) in October 2023. Subsequently, she developed bilateral eyelid ptosis, incomplete eye closure, dysarthria, dysphagia, limb weakness, difficulty chewing, and neck weakness. Worsening symptoms and dyspnea developed following a respiratory infection. Upon presenting to the emergency department, where she was diagnosed with MG based on a positive RNS test and neostigmine test. Serological testing demonstrated elevated AChR-Ab levels (>80 nmol/L). Thymus CT revealed probable incomplete thymic involution. At the time of presentation, her QMG and MG-ADL scores were 21 and 19, respectively. During the acute phase, she was treated with intravenous immunoglobulin (IVIg, 400 mg/kg/d for 5 d) and pyridostigmine (180 mg/d), resulting in partial improvement in limb weakness and swallowing function (QMG and MG-ADL scores decreased to 15 and 14, respectively). The patient also reported persistent dry eyes and dry mouth for over a year. Moreover, laboratory tests revealed a positive ANA (titer 1:320), along with elevated anti-SSA (2+) and anti-Ro52 (3+) antibodies. In addition, the positive results from the Schirmer’s I test and the tear film rupture test, which indicated a breakup time of 4 seconds in the left eye, also supported the diagnosis of Sjögren’s syndrome. Furthermore, salivary scintigraphy showed delayed uptake and reduced concentration in the patient with unstimulated total salivary flow (0.63 mL/15 min). Based on these findings, she was diagnosed with SS by the consulting rheumatologist according to the 2002 American-European Consensus Group (AECG) criteria. Telitacicept (160 mg/week) and low-dose prednisone (20 mg/d) were initiated for immunosuppressive therapy. Two weeks post-treatment, her dyspnea resolved, and other symptoms significantly improved, with QMG and MG-ADL scores decreasing to 11 and 8, respectively. Six weeks later, her QMG and MG-ADL scores improved to 1 and 0, respectively (**Figure 1**). Serological reexamination in July 2024 showed a reduction in AChR-Ab levels to 10.30 nmol/L. She has maintained MSE for one year, with significant relief from dry eyes and dry mouth symptoms. The prednisone dose was tapered to 5 mg/d and has been maintained to date (**Figure 2**).

### 2.3 Case 3

A 29-year-old man with MG was admitted to our hospital in February 2024. He presented with fluctuating ptosis that had begun four months earlier. He had been treated with pyridostigmine (180 mg/d) at a local hospital. Serological immune testing revealed elevated for AChR-Ab (>80 nmol/L) and titin-Ab (titer 1:300). Imaging confirmed the presence of a thymoma (AB type), and he subsequently underwent thymectomy. Despite surgery, his symptoms progressively worsened and included fluctuating dysarthria, dysphagia, limb muscle weakness, exertional dyspnea, diplopia, and gait instability. He received IVIg (400 mg/kg/d for 5 d) and prednisone (30 mg/d). After that, we found some abnormal serological testing results such as positive ANA (titer 1:100), anti-SSA (+), anti-Ro52 (2+) and anti-mitochondrial M2 antibodies (AMA-M2, 2+). In conjunction with 6-month dry eye symptoms (artificial tear-dependent), asymmetric corneal staining (right eye: 4; left eye: 1, based on the van Bijsterveld scale), and a labial gland biopsy that revealed focal lymphocytic infiltration with lymphocytes more than 50 cells per foci, the diagnosis of SS was established according to the 2002 AECG criteria. After IVIg treatment, his QMG and MG-ADL scores were 23 and 15, respectively. Because of the presence of multiple immune disorders, he was prescribed telitacicept (160 mg/week), pyridostigmine (240 mg/d), and prednisone (30 mg/d). After four weeks, his symptoms significantly improved, with complete resolution of diplopia, incomplete eyelid closure, and dyspnea, resulting in a QMG score of 12 and an MG-ADL score of 9. After eight weeks, he experienced marked improvement in limb weakness and regained the ability to ambulate, chew, and swallow, achieving a QMG score of 3 and an MG-ADL score of 1. Two months after starting telitacicept, he achieved MSE and has maintained this status for eight months (**Figure 1**). Moreover, his prednisone dose was gradually tapered from 30 mg/d to 5 mg/d over a 20-week period beginning with telitacicept initiation and has remained stable since (**Figure 2**).

## 3 Discussion

This study reports three cases of coexisting MG and CTD: one case associated with SLE and two with coexisting SS. All three patients showed marked clinical improvement after telitacicept treatment, achieving MSE while reducing prednisone doses. Simultaneously, the clinical symptoms of CTD were well controlled. Recent studies on the coexistence of ADs and MG have earned significant attention due to their shared mechanisms of immune dysfunction mechanisms (8). Similar to MG, CTD are multisystem autoimmune disorders characterized by diverse manifestations resulting from various autoantibodies that drive chronic inflammation. Increasing evidence underscores the pivotal role of B cells in the pathogenesis of both MG and CTD, suggesting overlapping pathogenic mechanisms. Genetic factors, such as human leukocyte antigen (HLA), contribute to MG susceptibility and are similarly implicated in other ADs (9, 10). Most MG-associated genes identified to date are involved in immune responses, a pattern observed in nearly all ADs (11). Thymic involvement is detected in up to 75% of patients with MG, with approximately 15% having an underlying thymoma and the remainder presenting with thymic hyperplasia (12). Thymic abnormalities are hypothesized to cause T-cell loss of self-tolerance and polyclonal B-cell activation, resulting in excessive autoantibody production and a predisposition to CTD. The shared features and distinct characteristics of these diseases pose challenges in managing these comorbidities, as no clear guidelines currently exist for their treatment.

B cells play a critical role in autoimmune diseases through antibody production, antigen presentation, pro-inflammatory cytokine secretion, ectopic germinal center formation and maintenance, and regulation of T-cell activation (13). Given the significance of B cells in AD pathogenesis, therapies targeting B cell depletion or inhibition represent a promising approach to restoring immune balance and achieving clinical improvement. Rituximab is a chimeric anti-CD20 monoclonal, which depletes immature, naive and memory B cells, resulting in reduced production of pathogenic antibodies. However, the limited efficacy of rituximab against AchR-MG may be due to the fact that CD20 is absent on the surface of long-lived plasma cells, the main source of AchR antibody production (14). Additionally, previous studies have demonstrated a delay of 6–8 weeks in the effects of rituximab for patients with MG (15, 16). Telitacicept, a novel recombinant fusion protein comprising the extracellular structural domain of a transmembrane activator and calcium modulator and cyclophilin ligand interactor, has been developed to treat B cell-mediated ADs (7). By binding to the BLyS and APRIL, and dually suppressing its activity, telitacicept not only inhibits the differentiation and survival of mature B cells but also prevents long-lived plasma cells from producing pathogenic antibodies (17, 18). Telitacicept has demonstrated significant efficacy in treating active SLE and has been approved for this indication (19). Given the role of B cells in the mechanism of pathogenicity of MG, it is speculated that telitacicept may also be effective in treating MG, as both are B cell-mediated autoimmune diseases. Moreover, a recent open-label, randomized, multicenter phase 2 clinical study involving 29 adult patients has demonstrated that telitacicept offers rapid clinical improvement and good tolerability (20). Thus, telitacicept may be a suitable double-targeted therapy for patients with co-occurring MG and CTD. In the present study, all three patients with MG-CTD achieved satisfactory QMG reduction following telitacicept treatment. During treatment, their immunoglobulin (IgA, IgG, and IgM) levels remained above the lower limit of normal, and no adverse events were observed. The favorable clinical responses and good tolerability imply the potential of telitacicept for treating MG-CTD. All three patients achieved MSE within 4–7 weeks of telitacicept administration and remained stable during six months of follow-up. Additionally, the prednisone dosage was reduced. These findings suggest that telitacicept may be a promising treatment option for patients with MG-CTD.

## 4 Conclusion

Our case reports present preliminary evidence supporting telitacicept as an effective and double-target therapy for the management of MG-CTD. However, our case reports only included three patients, the conclusion should be cautiously interpreted. The study has limitations including a small sample size, lack of controls and limited follow-up time. Therefore, large-scale randomized controlled trials with long follow-up are necessary in the future to provide robust evidence supporting the benefits of telitacicept in MG-CTD.

## Data Availability

The original contributions presented in the study are included in the article/Supplementary Material. Further inquiries can be directed to the corresponding author.
